# Revisiting the Role of Clathrin-Mediated Endoytosis in Synaptic Vesicle Recycling

**DOI:** 10.3389/fncel.2018.00027

**Published:** 2018-02-06

**Authors:** Ira Milosevic

**Affiliations:** Synaptic Vesicle Dynamics Group, European Neuroscience Institute (ENI), University Medical Center Göttingen (UMG), Göttingen, Germany

**Keywords:** endocytosis, endosomes, synaptic transmission, dyanmin, endophilin, clathrin, synaptic vesicle size, fast endocytosis

## Abstract

Without robust mechanisms to efficiently form new synaptic vesicles (SVs), the tens to hundreds of SVs typically present at the neuronal synapse would be rapidly used up, even at modest levels of neuronal activity. SV recycling is thus critical for synaptic physiology and proper function of sensory and nervous systems. Yet, more than four decades after it was originally proposed that the SVs are formed and recycled locally at the presynaptic terminals, the mechanisms of endocytic processes at the synapse are heavily debated. Clathrin-mediated endocytosis, a type of endocytosis that capitalizes on the clathrin coat, a number of adaptor and accessory proteins, and the GTPase dynamin, is well understood, while the contributions of clathrin-independent fast endocytosis, kiss-and-run, bulk endocytosis and ultrafast endocytosis are still being evaluated. This review article revisits and summarizes the current knowledge on the SV reformation with a focus on clathrin-mediated endocytosis, and it discusses the modes of SV formation from endosome-like structures at the synapse. Given the importance of this topic, future advances in this active field are expected to contribute to better comprehension of neurotransmission, and to have general implications for neuroscience and medicine.

The core of intracellular communication in sensory and nervous systems is achieved through neurotransmission, a process that transmits signals from one nerve cell to another. It is initiated by a short-lived raise and fall of electrical membrane potential (action potential) of a neuron, which allows calcium entry in a presynaptic terminal and consequent fusion of synaptic vesicles (SVs) with the plasma membrane, resulting in neurotransmitter release. SVs are can store and secrete various neurotransmitters, and are involved in the fast, point-to-point signaling across synapses. Notably, the existence of SVs is fundamental for maintaining key neuronal properties, including quantal release, modulation and directionality of synaptic signals. Due to a small size of presynaptic terminals in the brain, the number of SVs is limited, and SVs would be rapidly used up without efficient SV re-formation mechanisms. Moreover, synapses rely on the controlled recycling of SV membrane and proteins after every fusion event to maintain the net surface area and ensure for the repeated rounds of vesicle fusion, irrespectively of any demand enforced by neuronal activity (Murthy and De Camilli, 2003; McMahon and Boucrot, 2011; Saheki and De Camilli, 2012). Defects in SV recycling processes result in impaired neuronal function and neurodegeneration (Milosevic et al., 2011; Saheki and De Camilli, 2012; Murdoch et al., 2016).

It is known for over 40 years that the SVs are formed and recycled locally at the presynaptic terminal (Ceccarelli et al., 1973; Heuser and Reese, 1973). Nevertheless, the precise mechanisms of SV recycling still remain unclear (Kokotos and Cousin, 2015; Soykan et al., 2016; Watanabe and Boucrot, 2017). SVs have a well-defined protein composition and are uniquely small and homogeneous in size, suggesting that precise mechanisms are in place to shape and fission them. Five mechanisms for vesicle endocytosis at the synapse have been discussed to date: clathrin-mediated endocytosis (Heuser and Reese, 1973; Murthy and De Camilli, 2003; Logiudice et al., 2009), bulk endocytosis (Miller and Heuser, 1984; Holt et al., 2003; Wu and Wu, 2007), clathrin-independent fast endocytosis (von Gersdorff and Matthews, 1994; Boucrot et al., 2015; Delvendahl et al., 2016), ultrafast endocytosis (Watanabe et al., 2013a,b; reviewed in Watanabe and Boucrot, 2017) and direct SV reformation through fast closure of a transient fusion pore (kiss-and-run; Gandhi and Stevens, 2003; Zhang et al., 2009).

## Biogenesis and Recycling of Synaptic Vesicles at the Neuronal Synapse

**Figure 1:**
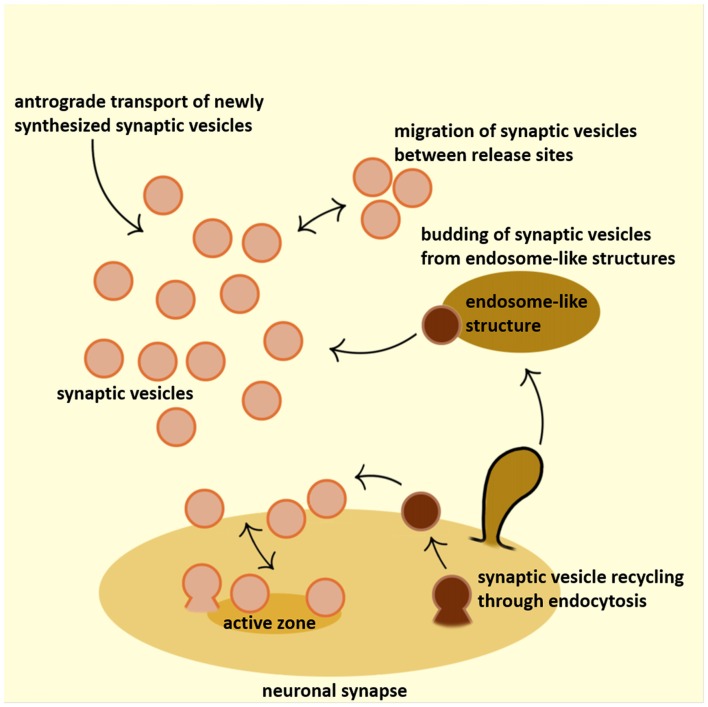
Schematic representation of the synaptic vesicle (SV) dynamics at the presynaptic terminal. SVs that accumulate at the presynaptic terminal originate from various sources: they are either actively transported to the synapses or recycled locally, either in the close proximity to the active zone (periactive zone) or budding from endosome-like structures. Furthermore, significant exchange of SVs happens between adjacent synapses. Active zone is depicted in dark yellow, SVs in orange, clathrin coated vesicles (CCVs) in dark brown and endosome-like structure in light-brown color.

The classical model of clathrin-mediated endocytic recycling at the presynaptic terminal implies that the newly retrieved and uncoated vesicles fuse with cisternae/endosome-like structures (Heuser and Reese, 1973). However, it has also been suggested that SVs can be derived directly from the uncoating of endocytic clathrin-coated vesicles (Takei et al., 1996). This latter hypothesis is consistent with the small and homogenous size of clathrin-coated vesicles, which are roughly the same size as SVs, with similar protein composition (apart from the clathrin coat proteins) and with the fast speed of SV recycling (as detailed below). In addition, several studies have suggested that an individual SV can take an extracellular tracer by a single endocytic event and release such tracer by a subsequent fusion event, without diluting it in the intermediate compartment (Ryan et al., 1997; Murthy and Stevens, 1998). In certain models (e.g., ribbon synapses of the inner hair cells that convert acoustic signals into action potentials in spiral ganglion neurons at rates of up to hundreds of Hz; Rutherford and Moser, 2016), or under certain conditions (e.g., strong neuronal stimulations triggered by high potassium), a large number of SVs undergoes exocytosis and collapse into the presynaptic plasma membrane almost simultaneously. As a consequence, the nerve terminals must be endowed with the robust capacities to maintain the net surface area and regenerate SVs with high efficiency and fidelity (Figure 1). It was also noted that, following the strong stimulations, endosome-like structures transiently accumulate at the synapses, and that new SVs are formed from those structures (Miller and Heuser, 1984; Pfeffer and Kelly, 1985). Such endosome-like structures are generally considered to be generated by bulk endocytosis, and not by gradual fusion of uncoated vesicles with the existing endosome-like structures (Figure 1; Murthy and De Camilli, 2003).

One remarkable feature of SVs is the comparative uniformity of their size at various synapses (~40 nm; van der Kloot, 1991). This homogenous size and efficient recycling mechanisms of SVs allow nerve cells to regulate neurotransmitter output primarily by regulating the number of SVs (quanta) that undergo exocytosis. Presently, it is unclear whether the size of the SV defines the amount of neurotransmitter that can be filled in the SVs, or vice versa. It was reported that SVs slightly expand in size as they fill with neurotransmitter (i.e., glutamate; Budzinski et al., 2009), thus suggesting that the neurotransmitter filling contributes to the SV size. Based on Budzinski et al. (2009), it may be possible to determine whether the SV is filled by neurotransmitter, or not, by carefully determining the vesicle size: this possibility is currently being tested. Curiously, only few cases have been reported where reformed SVs had on average larger or smaller size. For example, in the absence of dynamin 1, SVs were more variable in size, as determined by electron microscopy (EM) and by an increased amplitude of average miniature post-synaptic currents (i.e., a greater volume will result in an increased neurotransmitter content; Ferguson et al., 2007; Raimondi et al., 2011). The precise mechanisms that control the homogeneously small size of SVs partially break down also in the absence of some clathrin adaptor proteins, e.g., AP180 (Zhang et al., 1998), AP2 (Gu et al., 2013) and CALM (Petralia et al., 2012). While putative links between adaptor protein, protein sorting and SV morphology are becoming increasingly obvious, it is not clear how exactly this is achieved (Poudel and Bai, 2014). Interestingly, a small GTPase Rab5, a known regulator of endocytic vesicular transport, has also been found to maintain the uniform SVs size by preventing homotypic fusion between vesicles (Shimizu et al., 2003). Overall, given that the mechanisms controlling SV diameter are likely to be closely connected with the endocytic machinery that shapes and fissions them, understanding the role of proteins like AP180 or dynamin in regulating vesicle diameter is closely linked with the goal of understanding mechanisms of SV formation.

Furthermore, it is thought that even *de novo* formation of SVs at the presynaptic terminals occurs from components that have arrived at the synapse as a part of precursor membranes, and that newly synthesized proteins merge with recycling components into a single pool of proteins and membrane from which new vesicles are formed (as reviewed in Murthy and De Camilli, 2003). In general, the formation/recycling of new SVs is presently considered to be primarily a protein-mediated process, although the lipids have key regulatory roles (Saheki and De Camilli, 2012). Vesicle recycling via clathrin-mediated endocytosis (detailed below) is considered to be of vital importance and it is well understood (McMahon and Boucrot, 2011; Saheki and De Camilli, 2012). Molecular mechanisms that drive other endocytic forms that likely coexist with clathrin-mediated endocytosis at the synapse, namely kiss-and-run (transient SV fusion without the full collapse of the SV membrane; Del Castillo and Katz, 1955; Ceccarelli et al., 1973; Zhang et al., 2009), bulk endocytosis (the large addition of SV membranes within a short time, usually following the very intense stimulation, Heuser and Reese, 1973; Miller and Heuser, 1984; Holt et al., 2003; Paillart et al., 2003), clathrin-independent fast endocytosis (von Gersdorff and Matthews, 1994; Boucrot et al., 2015; Delvendahl et al., 2016) and/or ultrafast endocytosis (clathrin-independent membrane removal that occurs rapidly at the millisecond time scale, Watanabe et al., 2013a,b; Watanabe and Boucrot, 2017), are less understood, and are still being researched.

The clathrin-independent endocytic processes are not discussed in details here since several studious and informative reviews have been published not long ago (Kokotos and Cousin, 2015; Soykan et al., 2016; Watanabe and Boucrot, 2017). That said, the recent reports on the novel forms of clathrin-mediated endocytosis have sparked much discussion: the very existence of clathrin-mediated endocytic events at the presynaptic terminals are currently being questioned (Kononenko et al., 2014; Watanabe et al., 2014; Soykan et al., 2017). Notably, it has been realized that the majority of the experiments that have helped to establish clathrin-mediated endocytosis as a key event in SV formation at the presynaptic terminal were performed at room temperature (Watanabe et al., 2014; Soykan et al., 2017). Two groups have recently proposed that the clathrin-mediated endocytic events at the synaptic plasma membrane do not occur, or their frequency is largely reduced, when experiments are performed at the physiological temperatures (i.e., 37°C; Watanabe et al., 2014; Soykan et al., 2017). Follow-up experiments that address the temperature sensitivity of clathrin-mediated endocytosis at the synapse are now been performed by several independent groups in different model systems. The results so far suggest no role, or only partial role, for clathrin-mediated endocytosis in SV reformation at the plasma membrane (Kononenko et al., 2014; Nicholson-Fish et al., 2015; Delvendahl et al., 2016). Given that the latter studies question over three decades of research on clathrin-mediated endocytosis, and that some of them do not exclude the contribution of clathrin-mediated endocytosis at the latter time points after plasma membrane chunks have been already internalized, future work is expected to bring more clarity in the ongoing debate.

Remarkably, the occurrence of fast and slow endocytic forms has been observed a while ago by electrophysiological studies using membrane capacitance measurements, both in neurons (von Gersdorff and Matthews, 1994; Smith et al., 2008) and in neurosecretory chromafin cells (Artalejo et al., 1995). In particular, von Gersdorff and Matthews (1994) reported a fast endocytic form (with a time constant of 1–2 s) after a short stimulus, while a slower form of endocytosis (with a time constant of 10–20 s) is observed after a strong stimulation. These authors have also suggested that the fast form is clathrin-independent, and the slow form is clathrin-dependent (von Gersdorff and Matthews, 1994; Smith et al., 2008). The recent study by Delvendahl et al. (2016) has showed that hippocampal nerve terminals are indeed capable of fast endocytosis (with time constant of 0.4 s) after one action potential at 37°C: this fast endocytic form was temperature sensitive, clathrin-independent and dynamin- and actin-dependent (Delvendahl et al., 2016). Overall, the fast endocytosis observed after a short and weak stimulation (corresponding to one or few action potentials, as reported by von Gersdorff and Matthews, 1994; Delvendahl et al., 2016) may be a form of kiss-and-run, or ultrafast endocytosis proposed by Watanabe et al. (2013a,b, 2014). In contrast, the longer and stronger stimulations (corresponding to many action potentials) result in fast internalization of larger plasma membrane surface, and may represent bulk endocytosis. The slow endocytic form with time constants over 5–10 s most likely represents clathrin-mediated endocytosis.

Besides temperature, the maturity of a presynaptic terminal is also important for the endocytic process. Older, more mature, nerve terminals have faster endocytosis as well as the higher capacity for endocytosis after each round of exocytosis, most likely since these mature nerve terminals have higher levels of endocytic proteins available for the fast endocytic processes (Renden and von Gersdorff, 2007).

Altogether, revealing the molecular mechanisms responsible for the biogenesis of SVs and maintenance of quantal size is still one of the central question in neurobiology and neurophysiology. It is anticipated that, due of the fundamental nature of this questions, the advances in this area will deepen the understanding of both synapse and brain functions.

## Vesicle Recycling via Clathrin-Dependent Endocytosis

Clathrin-dependent endocytosis at the synapse actually represents a specialized form of the housekeeping membrane trafficking that occurs in all cells. The most common mechanism of vesicle formation is vesicle budding mediated by protein complexes/coats (e.g., COPI, COPII, clathrin; Bonifacino and Glick, 2004; Robinson, 2015; Dacks and Robinson, 2017). Coat proteins contribute to vesicle formation in two ways: first by capturing specific protein components known as membrane cargo that is to be incorporated into the newly-formed vesicles, and second, by stabilizing and/or inducing membrane curvature (Robinson, 2015).

Ample evidence collected over the past four decades implicates the clathrin coat and clathrin-mediated endocytosis in recycling of SV membrane after each round of release (Heuser and Reese, 1973; Koenig and Ikeda, 1989; Murthy and De Camilli, 2003; Augustine et al., 2006; Heerssen et al., 2008; Kasprowicz et al., 2008; Saheki and De Camilli, 2012). The high amounts of clathrin are present in the CNS where clathrin is enriched at the synapses (Maycox et al., 1992; Takei et al., 1995). Some of the key clathrin adaptor proteins like AP2 are also concentrated at the synapse (Gu et al., 2008). Clathrin-coated pits and vesicles are commonly observed by EM at the presynaptic terminals: Their number increases dramatically during the recovery phase after neuronal stimulation when endocytic recycling is promoted (Heuser and Reese, 1973), or when endocytic process is impaired by experimental manipulations that inhibit vesicle fission and recycling (Takei et al., 1995; Shupliakov et al., 1997; Cremona et al., 1999; Gad et al., 2000; Yamashita et al., 2005; Newton et al., 2006; Ferguson et al., 2007; Hayashi et al., 2008; Milosevic et al., 2011). SV formation can be impaired by both acute and chronic perturbations of clathrin using microinjections, photoinactivation, genetic and chemical approaches (Morgan et al., 2000; Granseth et al., 2006; Heerssen et al., 2008; Kasprowicz et al., 2008; Sato et al., 2009). Lastly, membrane proteins (cargo) in clathrin-coated vesicles purified from brain are quite similar to the membrane protein composition of SVs (Maycox et al., 1992; Pfeffer and Kelly, 1985; Blondeau et al., 2004). Actually, the preferred source of clathrin-coated vesicles or clathrin coats for the structural and biochemical studies is the brain tissue due to the abundance of clathrin at the synapses (Kirchhausen and Harrison, 1981). Thus, in my opinion, this form of endocytosis at the synapse will continue to be considered as a key step in the (re)formation of new SVs. Specifically, I expect that the clathrin coat machinery may have a role in breaking down the endosome-like structures formed by (ultra)fast endocytosis into the new functional SVs.

## Key Mechanistic Aspects of Clathrin-Dependent Endocytosis

**Figure 2:**
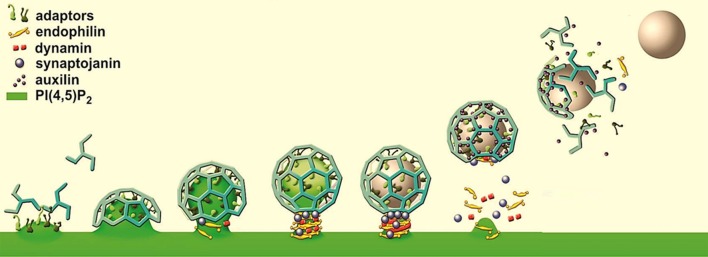
Model of clathrin-mediated endocytosis, a form of endocytosis that builds on the clathrin coat, the GTPase dynamin, and a variety of accessory factors, like clathrin adaptors, BAR proteins (e.g., endophilin) and lipid phosphatases (e.g., synaptojanin-1).

The classic view of SV fusion at the synapse postulates that the membrane and proteins of fused SVs are adding into the presynaptic plasma membrane, and are subsequently recovered by clathrin-mediated endocytosis (Rizzoli and Jahn, 2007). The key steps of clathrin-mediated endocytosis are well comprehended (Figure 2; Kirchhausen, 2000; Conner and Schmid, 2003; Milosevic et al., 2011; Saheki and De Camilli, 2012). The process starts with the recruitment of the clathrin adaptors by the plasmalemmal phosphatidylinositol(4,5)-bisphosphate [PI(4,5)P_2_] and cytosolic domains of SV proteins (cargo). Adaptor binding initiates recruitment of clathrin light and heavy chains in a form of triskelia that polymerize in the distinctive lattice, resulting in the formation of a deeply invaginated coated pit. The fission of such pit is mediated by dynamin, the GTPase that assembles in a collar-like structure at the pit neck. After the fission action of dynamin, a free coated vesicle can rapidly lose its coat through the action of synaptojanin-1, a phosphatase that hydrolyzes PI(4,5)P_2_ to promote the dissociation of clathrin adaptors, and the ATPase Hsc70 and its cofactor auxilin (to disassemble clathrin). Further, numerous studies have revealed that this core machinery is controlled by a variety of accessory factors, whose function, in most cases, is still poorly understood (Legendre-Guillemin et al., 2004; Itoh and De Camilli, 2006; Keyel et al., 2006; Jung and Haucke, 2007; Saheki and De Camilli, 2012). In particular, a superfamily of Bin/Amphiphysin/RVS (BAR) domain-containing proteins has an essential role by detecting curvature of a particular shape or size and inducing membrane curvature *de novo*, in order to recruit cytosolic factors to the curved membranes, and to stabilize existing curvature (Frost et al., 2008, 2009; Saheki and De Camilli, 2012).

Cell-type-specific peculiarities of clalthrin-mediated endocytic process are thought to exist and to be mediated primarily by accessory factors. Accessory factors fulfill several roles, including recruitment of cargo to the endocytic pits, modification of the lipid bilayer composition, membrane curvature generation and stabilization, signaling, coordination of bud formation with the nucleation and dynamic nature of the actin cytoskeleton, et cetera (McMahon and Boucrot, 2011; Saheki and De Camilli, 2012; Robinson, 2015). Many of these accessory factors and sequence of action are presently heavily investigated (e.g., Taylor et al., 2011, 2012). The relationship of various endocytic factors with each other and with actin polymerization is best described in yeast (Kaksonen et al., 2006). At the endocytic sites in yeast where a tubular invagination of the plasma membrane eventually pinches-off, actin plays a vital role. Yet, in spite of a rather good conservation between species, there are key differences in endocytic mechanisms between yeast and higher eukaryotes. Notably, the role of actin in clathrin-mediated endocytosis in various cell types is still debated. It has been reported that the mutations in a number of genes that affect actin function in fruit fly, such as intersectin (Marie et al., 2004; Koh et al., 2007) and a Bin/Amphiphysin/RVS (BAR) protein *nervous wreck* (Rodal et al., 2008), impair SV recycling. Clathrin-mediated recycling of SVs was itself proposed to be actin-dependent at lamprey giant axon synapses (Shupliakov et al., 2002; Bourne et al., 2006), but actin-independent at the CNS synapses (Sankaranarayanan et al., 2003). It is however important to note that the experiments reported in Sankaranarayanan et al. (2003) were performed at the room temperature, whereas more recent studies have shown that actin-dependent ultrafast endocytosis occurs only near physiological temperatures (Watanabe et al., 2013b, 2014; Soykan et al., 2017). Thus, actin may nevertheless have a role in SV reformation at the physiological temperatures.

Interestingly, in the fibroblasts without the GTPase dynamin, clathrin-coated pits become arrested at the tip of long and narrow actin-embedded tubules that are reminiscent of the tubular invaginations observed at actin patches in yeast (Kaksonen et al., 2006; Wu et al., 2010). In addition, several proteins, like N-WASP, CD2AP, Arp2/3, endophilin-A and F-actin components are the mammalian homologs of yeast actin patch proteins. Therefore, it seems that the absence of dynamin unmasks putatively evolutionary-conserved close relationship between clathrin-mediated endocytosis and yeast endocytosis. Building on this and similar examples, it is expected that the precise mechanistic understanding of processes occurring during clathrin-mediated endocytosis will assist the present comprehensive efforts to elucidate the mechanisms of presynaptic membrane trafficking pathways and the activity-dependence in the formation of SVs.

## Specialization of Endocytosis Mechanisms in Synaptic Vesicle Recycling

Several modes of endocytic trafficking (see above) were described to co-exist and operate concurrently in the same cell. For example, clathrin-dependent endocytosis at the synapse represents a highly specialized endocytic form that occurs at the surface of all cells. The recognition of novel endocytic pathway as a distinct, discrete process is primarily based on the presence of defined lipids, cargo proteins and their regulators. The internalization route and cargo specificity seem to be determined by accessory factors that mediate cargo enrichment, the choice of the coat and coat-associated protein assembly and even a scission mechanism (Kumari et al., 2010). Both proteins and membrane parameters can influence and diversify endocytic process, as there are several diverse physical and biochemical principles behind the vesicle formation. The detailed mechanisms and kinetics are known only for the best characterized modes, such as clathrin-mediated endocytic process and, in yeast, endocytosis driven by actin-dependent forces (as detailed above).

Curiously, analysis of synapses with partial dynamin function (synapses of dynamin 1 knock-out and dynamin 1 and 3 double knock-out (DKO) mice) revealed a strong accumulation of clathrin-coated pits with long tubular necks, indicating a substantial delay of the fission reaction (Ferguson et al., 2007; Hayashi et al., 2008; Milosevic et al., 2011; Raimondi et al., 2011). Here, clathrin-coated pit accumulation correlates well with a substantial accumulation of known accessory endocytic factors thought to act upstream of, or together with, dynamin in the fission of SVs from the plasma membrane (Milosevic et al., 2011; Raimondi et al., 2011). Remarkably, these observations reveal some similarities in the endocytic intermediates between neuronal and non-neuronal cells, which are in this case potentiated by deficient dynamin function. On the contrary, it seems that additional regulatory mechanisms exist in neuronal cells: the comparisons between two different neuron subtypes, glutamatergic and GABAergic neurons within the same primary cortical culture revealed that the accumulation of endocytic proteins is much more prominent in GABAergic neurons (Hayashi et al., 2008; Milosevic et al., 2011; Raimondi et al., 2011). A simple explanation is that the GABAergic neurons have a higher tonic activity levels which unmasks endocytic defects more strongly in these system. Understanding the basis of this differential endocytic behavior of neuronal sub-types is a topic of present research efforts, and will be relevant for comprehending SV recycling and synaptic function.

## Fate of Clathrin Coated Vesicles after Endocytosis

Clathrin coated vesicles (CCVs) swiftly shed their coat after fission through a synchronized action of synaptojanin-1 (recruited by endophilin-A, Milosevic et al., 2011), the J-doman kinase auxilin and the ATP-dependent chaperone Hsc70 (Rothman and Schmid, 1986; Saheki and De Camilli, 2012). Notably, auxilin binds the clathrin lattice in such a way that allows Hsc70 to bind clathrin’s C-terminal hydrophobic sequence, and produce a local distortion of the lattice, which is then stabilized by ATP hydrolysis (Xiao et al., 2006; Jiang et al., 2007). Thus, it is essential that auxilin is recruited to the CCV only after the fission is finalized, or else the actions of Hsc70 and auxilin would obstruct the clathrin coated pit formation. It is not yet clear how the clathrin coat assembly-disassembly cycle is regulated, and how the auxilin recruitment is timed. A likely mechanism points to the action of synaptojanin-1 phosphatase the lipid modification after fission (Milosevic et al., 2011).

After uncoating, newly formed vesicles may fuse with endosomal compartment as proposed already by the first model of endocytic recycling (Heuser and Reese, 1973). However, it was also suggested that SVs can be derived straight from the uncoated CCVs (Takei et al., 1996). Further, it is considered that even ex-novo formation of SVs occurs at the nerve terminals from components that have reached the synapse as part of precursor membranes, and newly synthesized proteins merge with recycling components into a single pool of proteins and membrane from which new vesicles are formed (Murthy and De Camilli, 2003). Continued investigations along this line are required to unequivocally answer this point.

## Synaptic Vesicle Formation from Endosome-Like Structures

A dynamic population of endosome-like structures after neuronal stimulation, generated either by strong and prolonged stimulation through bulk endocytosis, or weak and brief stimulation by (ultra)fast endocytosis, opens the question whether these structures are related to classical (conventional) endosomes. Traditionally, endosomes are viewed as relatively stable organelles that serve as the first molecular sorting stations for endocytic traffic (Helenius et al., 1983; Wandinger-Ness and Zerial, 2014). They form a fascinating interconnected network of hundreds of vesicles and tubules that have numerous roles in trafficking of various cargoes and cellular signaling. Classical endosomes are undoubtedly present at the presynaptic terminals (Wucherpfennig et al., 2003). In contrast, endosome-like structures formed at the synapse after neuronal stimulation have a very transient nature: they form rapidly and then disappear. They are likely the most transient, most heterogeneous and certainly the least understood presynaptic structures, yet central to membrane trafficking at the synapse given that they control both the SV recycling and degradation of membrane proteins (Bonifacino and Glick, 2004; Saheki and De Camilli, 2012; Jähne et al., 2015).

Despite much knowledge on classical endosomes that has been gathered in the past three decades (Wandinger-Ness and Zerial, 2014), little is known about endosomes and endosome-like structures at the synapse, and the way they are linked to SV recycling. Specifically, it is not clear whether such endosomes/endosome-like structures maintain the plasma membrane properties (e.g., PI(4,5)P_2_ presence) for a brief time frame and take part in SV reformation, or selectively in housekeeping recycling and/or signaling pathways. Several reports indicate that endososomal compartments may play a role as sorting stations for SV proteins (Voglmaier et al., 2006; Opazo et al., 2010; Uytterhoeven et al., 2011; Jähne et al., 2015). In addition, the relationship between classical endosomal compartments implicated in house-keeping functions (e.g., receptor-mediated endocytosis) and endosome-like structures (also called vacuoles) that form in response to strong stimuli is not well understood.

Additional complexity in this field rises also from an absence of consensus on definitions and markers for various synaptic endosomal compartments (Jähne et al., 2015). As aforementioned, synaptic endosomes are seen either as stable organelles that are permanently present in the presynapse, or as short-lived intermediates resulting from homotypic fusion of smaller vesicles, or intermediates in SV recycling, arising from the endocytosis of large plasma membrane chunks. Besides, the data on the role of endosomes at the synapse are limited and controversial: while some researchers have suggested that endosomes are involved in the sorting of SV proteins, others deny this possibility (Jähne et al., 2015). Nevertheless, to date it is well-established in several species that neuronal synapses contain several molecular components of endosomes, for example proteins Rab5, Rab7, Rab11, amyloid precursor protein-like (APPL) and lipid phosphatidylinositol-3-phosphate (PI3P; Wucherpfennig et al., 2003; Takamori et al., 2006; Rodal et al., 2008; Brown et al., 2009). Even if the role of Rab proteins in SV recycling traffic remains debated, it seems that, with the exception for Rab5 marker, endosomal compartments at the synapse do not expand in response to neuronal stimulation. Notably, Rab5 manipulations (dominant-negative mutants in mammalian neurons and invertebrate knock-outs) result in defective SV recycling (de Hoop et al., 1994; Fischer von Mollard et al., 1994; Shimizu et al., 2003). However, Rab5 may be an exception here given that it seems to be a common component of newly internalized vesicles from the plasma membrane (Zoncu et al., 2009) and that in its absence the size of SVs increases (Shimizu et al., 2003), although it is primarily considered as a marker of well-defined early endosomes.

In conclusion, many outstanding questions remain in the field of synaptic endosomal compartments and their role in SV recycling. Thus, it is not surprising that the synaptic endosomal compartments, both in resting and strongly stimulated nerve terminals where endosome-like structures are considerably expanded, are presently a topic of intensive research studies.

## Modes of Synaptic Vesicle Formation from Endosome-Like Structures

**Figure 3:**
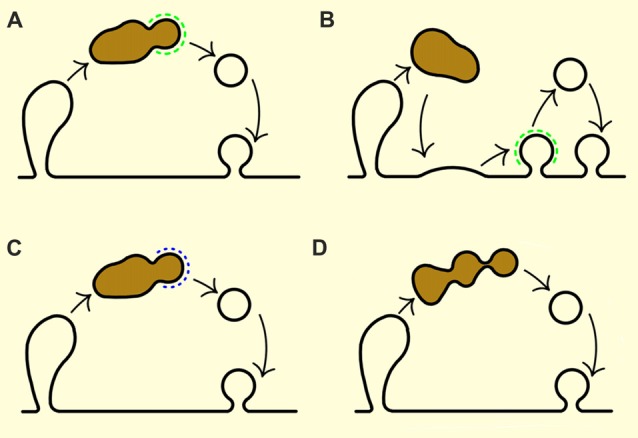
Modes of SV recovery from endosome-like structures. **(A)** Clathrin-mediated budding. **(B)** Fusion of endosome-like structures with the plasma membrane (a sort of homotypic fusion of bulk internalized plasma membrane fragments) followed by subsequent recovery of SV proteins from the plasma membrane by clathrin-mediated endocytosis. **(C)** Budding by clathrin-independent mechanisms but mediated by some coat proteins. **(D)** Tubulation followed by tubule fragmentation without classic coat proteins. Endosome-like structures are depicted in brown and clathin coat in green. Black lines outline the membrane contours, and blue dotted line represents clathrin-independent coat.

Despite endosome-like structures that appear after neuronal stimulation have been observed for a while, it is currently not known how these structures convert to new SVs eventually. Here, I summarize the latest discoveries and outline the four main scenarios that can explain the conversion of endosome-like structures to SVs. Briefly, SVs may re-form from endosome-like structures by: (i) clathrin-mediated mechanism directly from endosome-like structures; (ii) clathrin-mediated mechanism after endosome-like structure back-fuses with the plasma membrane; (iii) a novel lipid- or protein-mediated means that are independent of classical coats; and (iv) tubulation followed by tubule fragmentation. For a schematic representation of these four scenarios, see Figure 3.

First, new SVs may be formed from endosome-like structures generated by (ultra)fast endocytosis through clathrin-mediated budding process that occurs at them (as originally suggested by Heuser and Reese, 1973; Takei et al., 1996; Figure 3A). Indeed, a number of studies clearly report the occurrence of clathrin-coated pits on endosome-like structures at the synapse, including the recent work of Kononenko et al. (2014) and Watanabe et al. (2014). Notably, knock-down of clathrin seems to arrests SV formation from endosomes in hippocampal presynaptic terminals (Kononenko et al., 2014; Watanabe et al., 2014), and the ablation, or knock-down, of AP2 or adaptor protein 1 (AP1)/adaptor protein 3 (AP3) arrests SV formation from endosomes (Cheung and Cousin, 2012; Kononenko et al., 2014). Yet surprisingly, several careful EM and EM tomography studies performed on the intact nerve terminals of mice without dynamin 1, or mice without dynamin 1 and 3, have found that almost all clathrin coated pits observed are directly connected to the plasma membrane, also when they seem to be budding from endosome-like structures in the single EM sections (Ferguson et al., 2007; Hayashi et al., 2008; Raimondi et al., 2011; Wu et al., 2014). The latter studies suggest that most clathrin-mediated endocytosis happens on the plasma membrane, rather than on the endosome-like structures.

Second, endosome-like structures, in particular the ones formed after strong stimulation, may fuse again with the plasma membrane in a manner of homotypic fusion of bulk-internalized plasma membrane fragments; this process is then followed by gradual SV formation from the plasma membrane by clathrin-mediated endocytosis (Figure 3B). Aforementioned tomographic studies of mammalian synapses from wild-type and dynamin mutants in which clathrin-mediated endocytosis is strongly impaired support this scenario in principle, since these models have revealed the exceptionally rare presence of clathrin buds on endosome-like structures despite new SVs being present also on these mutant synapses (Ferguson et al., 2007; Hayashi et al., 2008; Raimondi et al., 2011; Wu et al., 2014).

Third, the new SVs may be re-formed from endosome-like structures through clathrin-independent budding process that is nonetheless mediated by some type of coat (Figure 3C). The occurrence of clathrin-independent endocytic mechanisms (detailed below) has been brought up by several studies in worms (Gu et al., 2008; Sato et al., 2009). It is presently unclear which other coat could mimic the role of clathin coat in this process: possibilities include AP-1 or AP3-based coats (Faúndez et al., 1998; Nakatsu et al., 2004; Kim and Ryan, 2009; Newell-Litwa et al., 2009; Glyvuk et al., 2010). For example, AP-3-based coat has been associated with the loading of specific cargo proteins into SVs (Blumstein et al., 2001; Newell-Litwa et al., 2009). Further, AP-3 coat appears to mediate clathrin-independent vesicle budding from endosomes in neurosecretory PC12 cell (Faúndez et al., 1998). Yet, though AP-3 and AP-1 have been implicated in the endocytic process at the synapse as well as SV recycling (Voglmaier et al., 2006; Glyvuk et al., 2010), they are not enriched at the synapses.

Fourth, SVs may re-form from endosome-like structures by a still unreported mechanism that may be either protein-, or lipid-mediated, but independently of classical proteinaceous coats (Kirchhausen, 2000; Graham, 2004; Saheki and De Camilli, 2012; Figure 3D). Wu et al. (2014) have proposed an existence of a SV recycling pathway that bypasses the requirement for dynamin 1 and 3 or clathrin, and that operates during strong stimulation. Here, tubuli originating from endosomal-like structures may be followed by tubuli fragmentation by lipid/protein factors (i.e., lipids may be metabolically modified to generate membrane asymmetry Graham, 2004; Bossard et al., 2007), or cytosolic proteins able to generate membrane curvature and thus act as non-conventional coats (Itoh and De Camilli, 2006). Namely, selective BAR proteins (e.g., endophilin-A) seem to be able fragment tubuli into small, non-coated vesicles *in vitro* (Gallop et al., 2006; Wang et al., 2009; Simunovic et al., 2017). However, such a mechanism may not be able to achieve the efficient sorting of all SV proteins, so the fidelity of SV reformation may be partially lost. Vesicles created in such way may need to undergo a new cycle of exocytosis that is followed by clathrin-mediated endocytosis in order to generate a functional SV with a well-defined protein composition and size.

## Synaptic Vesicle Can Form without Clathrin

The clathrin lattice consists of clathrin heavy and light chains: the heavy chains are needed for the formation of the lattice and therefore the endocytic function of clathrin, while the light chains have a regulatory function. Suppression of clathrin heavy chain expression has resulted in a consequent disappearance of the light chain (Hinrichsen et al., 2003). Clathrin is essential for development: the knock-out of the clathrin heavy chain gene produced embryonic lethality (Bazinet et al., 1993; Grant and Hirsh, 1999). Yet, it seems indispensable for the short-term survival of eukaryotic cells, based on data on its disruption in yeast (Lemmon and Jones, 1987), or in mammalian cells using knockdown-based approach (Motley et al., 2003; Granseth et al., 2006). Thus, the complete elimination of clathrin in neuronal cell could be achieved and it was the key test for the relevance of clathrin-independent endocytic process in the SV formation. When clathrin was deleted or inactivated in primary neuronal cultures, either by direct and indirect methods (including photoinactivation), a dramatic inhibition of SV formation was observed (Granseth et al., 2006; Heerssen et al., 2008; Kasprowicz et al., 2008). These studies thus support the strategic role of clathrin in SV recycling, and indicate a major role for clathrin-mediated endocytosis. Yet, the sequestration of other relevant factors into complexes with inactivated clathrin, or the possibility of unspecific protein damage by the reactive oxygen species generated by this approach cannot be ruled out completely. Curiously, clathrin knock-down studies led only to a partial inhibition of SV recycling, and there was no evidence of SV depletion under basal conditions (Granseth et al., 2006).

In the light of aforementioned studies and the well-accepted role of clathrin in SV formation, a study in worms by the Grant and Jorgensen laboratories produced an unexpected result (Sato et al., 2009). A temperature-sensitive clathrin mutant had a mutation in gene for clathrin heavy chain, which resulted in the addition of 22 amino acids to clathrin’s C-terminus and the severe reduction of clathrin levels. The remaining clathrin behaves abnormally (fails to assemble efficiently) upon a shift to the restrictive temperature. Yet, synapses of these mutant worms contained SVs (albeit of somewhat abnormal size) and had normal miniature synaptic current amplitudes and frequencies. Since kiss-and-run exo-endocytosis process postulates a preservation of vesicle structure rather than new vesicle generation, kiss-and-run endocytic mode would not be able to account for formation of SVs without clathrin. Further, this observation was also in line with two other studies in worms and in mammalian synapses showing that the clathrin adaptor protein 2 (AP2), the main endocytic adaptor for clathrin, is expendable for synaptic transmission (Gu et al., 2008; Kim and Ryan, 2009). Collectively, these findings raise questions about the essential nature of clathrin in SV recycling and call for further investigations.

## Synaptic Vesicles Can Form without Dynamin

Dynamin has an essential role in endocytic membrane fission events (Hinshaw, 2000; Ferguson and De Camilli, 2012). Different from invertebrates, mammals have three dynamin genes: dynamin 1, 2 and 3. The expression of dynamin 1 and dynamin 3 genes is the strongest in brain, while dynamin 2 shows a ubiquitous tissue distribution (Ferguson and De Camilli, 2012). In brain, dynamin 1 seems to be needed primarily in presynaptic vesicle recycling, while dynamin 3 was reported to have a postsynaptic function (Lu et al., 2007). The mandatory role of dynamin in endocytic fission of vesicle buds has been commonly accepted since the *shibire* mutation of *Drosophila* was mapped to the dynamin gene (Chen et al., 1991; van der Bliek and Meyerowitz, 1991). In this temperature-sensitive mutant, endocytosis sharply stops at the restrictive temperature (Koenig and Ikeda, 1989). A number of diverse studies have supported an fundamental role of dynamin in SV endocytosis as well as in clathrin-mediated endocytosis, including the expression of dominant negative dynamin mutants (van der Bliek et al., 1993), microinjections of antibodies or peptides that perturb dynamin interactions (Shupliakov et al., 1997), non-hydrolyzable analogs of GTP (Takei et al., 1995; Yamashita et al., 2005), pharmacological inhibition by dynasore (Newton et al., 2006). Yet, all key studies act by blocking dynamin’s actions, not by eliminating dynamin itself.

Curiously, several studies based on dynamin knock-out animals have challenged the essential role of dynamin. Mice without dynamin 1 appeared normal at birth, and survived up to 2 weeks (Ferguson et al., 2007), implying that the basic function of synapses is preserved without dynamin 1, which is the most abundantly expressed dynamin family member in the mammalian brain. Mice without dynamin 1 and 3 (dyanmin 1 and 3 together represent the majority of total brain dynamin) die at birth and SV endocytosis is dramatically impaired, yet, SVs can still form, and a subset of synapses show synaptic transmission (Raimondi et al., 2011; reviewed in Ferguson and De Camilli, 2012). These findings raise the question of potential compensatory roles of ubiquitously expressed dynamin 2, or given the relatively minor contribution of dynamin 2 to the total brain dynamin, they raise the possibility of dynamin-independent mechanisms of SV recycling (Ferguson and De Camilli, 2012).

Interestingly, the effect of dynamin absence seems much more severe in fruit flies (fruit fly has only one gene encoding dynamin): using acute photoinactivation of dynamin in *Drosophila melanogaster*, Kasprowicz et al. (2014) have shown that complete loss of dynamin function have entirely blocked membrane recycling and caused the buildup of large membrane-connected cisternae. The shRNA knockdown of dynamin-1 and dynamin 3 in hippocampal nerve terminals reported by Kononenko et al. (2014) reveals stronger phenotype than it is observed in dynamin 1 and 3 DKO synapse (Raimondi et al., 2011). Further, studies of membrane recycling after strong stimulation in dynamin 1 knock out, as well as in dynamin 1 and 3 DKO neurons, revealed the robust accumulation of endosome-like structures that are likely formed by bulk endocytosis (Wu et al., 2014). Given that these endosome-like structures can subsequently convert into SVs (Wu et al., 2014), the most likely explanation here is that synapses with partial dynamin function rely significantly on the bulk endocytosis to (re)form SVs. Such formation of SVs from endosome-like structures, however, seems to occur in a dynamin- and clathrin-independent manner (Wu et al., 2014).

## Synaptic Vesicles Can Form without Coat Proteins

Coats that assist vesicle formation share several common features, including the recruitment via small GTPases and acting as scaffolds in order to couple cargo selection to vesicle formation (Robinson, 2015; Dacks and Robinson, 2017). The discovery and characterization of several coats (COPI, COPII, clathrin) advocated that the spontaneous propensity of proteinaceous membrane-binding scaffolds to assemble into curved structures are main determinants in vesicle formation. However, additional mechanisms may also be relevant for the generation of highly curved membrane vesicles, like SVs. In addition, it would be advantageous for neuronal cells to have back-up mechanisms to generate SVs in a coat-independent fashion.

Curiously, it seems that new SVs can be generated at the synapses with aberrant dynamin function, but they are generally more heterogeneous in size than in controls (Ferguson et al., 2007; Wu et al., 2014). Consistent with an alternative mechanism of vesicle formation, binding of proteins with an intrinsic curvature to the membrane and/or an insertion of amphiphilic helices into the cytosolic leaflet of the membrane bilayer (Farsad and De Camilli, 2003; McMahon and Gallop, 2005), as well as an existence of membrane proteins that are able to generate asymmetry in the two membrane leaflets may also generate high curvatures (Shibata et al., 2006). Moreover, chemical modifications of the membrane bilayer, for example focal accumulation of diacylglycerol at the trans-Golgi network (Baron and Malhotra, 2002) or flippases (Graham, 2004) may assist curvature generation by mechanisms intrinsic to the bilayer. Curiously, studies in fruit fly have implicated diacylglycerol levels in SV endocytosis (Huang et al., 2004). Lastly, BAR proteins (e.g., endophilin-A) are also implicated in dynamin-independent fission of tubular membrane necks (Renard et al., 2015; Simunovic et al., 2016, 2017). Such protein scaffolds may bind the underlying tubuli to create a barrier that disables diffusion of lipids, while the elongation (stretching) of tubuli, assisted by motors (e.g., actin), may build local membrane tension until such membrane undergoes scission (Simunovic et al., 2016, 2017).

## Concluding Remarks

Endocytic processes and the mechanisms of SV recycling at the presynaptic sites are well investigated during the past decades, yet a lot remains unknown. Molecular mechanisms of (ultra)fast endocytic pathways that mediate the recapture of SV membrane after exocytosis are presently under intense investigation, and new breakthroughs along these lines are expected soon. The essential features of clathrin-mediated endocytic process at the synapse have been well understood, however, the question of its relevance at the physiological temperatures has recently been raised. I consider that clathrin-mediated endocytosis will persist in being considered a key step in the (re)formation of new SVs at the synapse. Specifically, in my opinion, the endosome-like structures formed by ultrafast endocytosis may maintain key features of plasma membrane (i.e., PI(4,5)P_2_ presence) for a brief time after internalization which allows them to utilize clathrin coat machinery to get broken into new functional SVs.

The dynamics and fate of endosome-like structures and newly endocytosed vesicles is also not well understood. It is enigmatic how the pace and flexibility of the SV recycling are controlled and maintained. The high speed of SV recycling and the small size of transient intermediate recycling forms hamper the progress here. The recent emergence of mouse models with defective endocytosis provides new tools: these models accumulate recycling intermediates at the synapse that are stable for longer periods (minutes to hours), in opposition to their lifetime of few milliseconds to seconds at the wild-type functional synapse (Ferguson et al., 2007; Milosevic et al., 2011; Raimondi et al., 2011). Furthermore, the current technological advances, such as super-resolution microscopy, EM tomography, fast sample stimulation with high-pressure freezing techniques (Kittelmann et al., 2013; Watanabe et al., 2013a,b), will allow the inspection of synaptic organelles within milliseconds of their formation, and will likely provide new insights. Taking into account the importance of the efficient SV recycling, it can be anticipated that new developments along these fronts will decisively advance the field of synaptic transmission, while having broad implications for neurophysiology and medicine.

## Author Contributions

The author confirms being the sole contributor of this work and approved it for publication.

## Conflict of Interest Statement

The author declares that the research was conducted in the absence of any commercial or financial relationships that could be construed as a potential conflict of interest.
